# The autophagy gene product BEC-1 supports normal aging and neurodevelopment in *Caenorhabditis elegans* III

**DOI:** 10.17912/micropub.biology.000101

**Published:** 2019-06-14

**Authors:** Nicholas Ashley, Andrea M Holgado

**Affiliations:** 1 St. Edward's University, Department of Biological Sciences, Austin, TX 78704, USA

**Figure 1.**
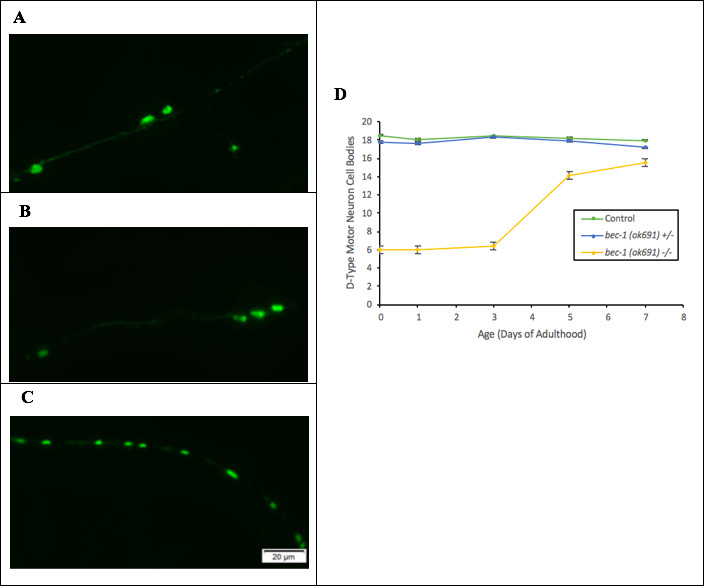
**Homozygous *bec-1(ok691)* mutants show a delay in the development of VD motor neurons.** (A-C) Representative fluorescent micrographs of D-type motor neuron expressing GFP in (A) control, (B) homozygous *bec-1(ok691)* mutant, and (C) heterozygous *bec-1(ok691)* mutant nematodes on day 5 of adulthood. (D) Quantification of D-type motor neuron cell bodies on days 0,1,3,5, and 7 of adulthood was recorded. Data plotted are mean ± 1 SEM, n=60. Statistical analysis was performed using a non-parametric Kruskal-Wallis (p< .001).

## Description

The loss of autophagy function in the motor cortex has been associated with progression of neurodegenerative symptoms in Parkinson’s disease (Kaila and Lang 2015; Fahn et al. 2004). To analyze possible effects of the *bec-1(ok691)* mutation on neuronal density, we followed the transgene *juIs76* as it produced GFP marked D-type motor neurons (Figure 1 A-C). Previous research has found lineage timing of GABAergic (VD) motor neuron differentiation in *C. elegans* to occur before animals reach adulthood (Jin et al., 1994). These studies show a delay in development of ventral D-type motor neurons in *bec-1(ok691)* homozygous mutants (Figure 1 D). Maturation and development of VD motor neurons to the levels of controls were seen on day 5 of adulthood in *bec-1(ok691)* homozygous mutants. However, our discovery in delayed lineage timing of VD motor neurons suggests a potential role of BEC-1 in neurodevelopment. This is consistent with findings in mouse models, where ortholog *Beclin 1* plays an essential role in cell differentiation during development (Cecconi and Levine, 2008). Instead of observing rapid neurodegeneration of VD motor neurons, resulting from the *bec-1(ok691)* mutation, we observed a rapid decrease in lifespan (Ashley and Holgado, 2019) as VD motor neurons were differentiated. This conclusion should be considered as preliminary as we have not verified by an alternative line of investigation (e.g., a second allele or transgene rescue) that the observed phenotypes are specific to *bec-1(ok691).*There is additional evidence that suggests autophagy’s role in mechanisms of cell editing in early developmental stages of *C. elegans* (Di Bartolomeo et al., 2010).

## Methods

Synchronizing:

Mixed stage nematodes grown on NGM plates at 20 °C were floated off using 1 mL of M9 reagent and collected in 1.5 mL tubes. Tubes containing animals were centrifuged at 9.3 × g for 1 minute. After centrifugation, the supernatant was discarded and the worm pellet was kept and treated with 1 mL of Alkaline Bleach (2.0% bleach (VWR), 0.5N NaOH) for 7 minutes at room temperature with occasional mixing. Once the 7-minute treatment concluded, bleached animals were centrifuged at 9. 3 × g for 2 minutes to collect eggs. Pelleted eggs were washed 3 times with 1 mL of M9 and centrifuged for 1 min. at 9.3 × g. After centrifugation, the supernatant was discarded and the pelleted eggs were suspended. Two drops of resuspended eggs were placed onto seeded NGM plates.

Neuron Cell Body Count:

Individuals were mounted on 2% agarose padded microscope slides in 2 drops of mineral oil. Using an Olympus fluorescent microscope (BX41), GFP positive neuronal cell bodies were counted on days 0,1, 3, 5, and 7 of adulthood. Neuron cell body count was reported as the average number of cell bodies per animal over time.

## Reagents

Strains CZ1200 and VC517 were obtained from the *C. elegans* Genetics Center. CZ1200 contains the transgene *juIs76*[*unc25p*::GFP] which drives the expression of GFP in d-type motor neurons. Strain AMH50 was produced in our laboratory by crossing CZ1200 with VC517 *bec-1(ok691)*/*nT1*[*qIs51*]. AMH50 possess the balanced lethal mutation *bec-1(ok691)*/*nT1*[*qIs51*] and the transgene *juIs76*, {*bec-1(ok691)*IV/*nT1*[*qIs51*](IV;V);*juIs76*[*unc-25p*::GFP] II}.
